# Comparing factor mixture modeling and conditional Gaussian mixture variational autoencoders for cognitive profile clustering

**DOI:** 10.3389/fpsyg.2025.1474292

**Published:** 2025-05-09

**Authors:** Matteo Orsoni, Sara Giovagnoli, Sara Garofalo, Noemi Mazzoni, Matilde Spinoso, Mariagrazia Benassi

**Affiliations:** Department of Psychology, University of Bologna, Bologna, Italy

**Keywords:** variational autoencoders, clustering, machine learning, cognitive profiles, factor mixture modeling

## Abstract

**Introduction:**

Understanding individual cognitive profiles is crucial for developing personalized educational interventions, as cognitive differences can significantly impact how students learn. While traditional methods like factor mixture modeling (FMM) have proven robust for identifying latent cognitive structures, recent advancements in deep learning may offer the potential to capture more intricate and complex cognitive patterns.

**Methods:**

This study compares FMM (specifically, FMM-1 and FMM-2 models using age as a covariate) with a Conditional Gaussian Mixture Variational Autoencoder (CGMVAE). The comparison utilizes six cognitive dimensions obtained from the PROFFILO assessment game.

**Results:**

The FMM-1 model, identified as the superior FMM solution, yielded two well-separated clusters (Silhouette score = 0.959). These clusters represent distinct average cognitive levels, with age significantly predicting class membership. In contrast, the CGMVAE identified ten more nuanced cognitive profiles, exhibiting clear developmental trajectories across different age groups. Notably, one dominant cluster (Cluster 9) showed an increase in representation from 44 to 54% with advancing age, indicating a normative developmental pattern. Other clusters displayed diverse profiles, ranging from subtle domain-specific strengths to atypical profiles characterized by significant deficits balanced by compensatory abilities.

**Discussion:**

These findings highlight a trade-off between the methodologies. FMM provides clear, interpretable groupings suitable for broad classification purposes. Conversely, CGMVAE reveals subtle, non-linear variations in cognitive profiles, potentially reflecting complex developmental pathways. Despite practical challenges associated with CGMVAE's complexity and potential cluster overlap, its capacity to uncover nuanced cognitive patterns demonstrates significant promise for informing the development of highly tailored educational strategies.

## 1 Introduction

Individual cognitive profiles, encompassing unique strengths and weaknesses in domains such as memory, attention, processing speed, and reasoning, play a pivotal role in shaping the learning process (Altun, 2016; Nesayan et al., 2018; Webster, 2002). Research indicates that personalized instructional strategies aligned with these cognitive differences can significantly enhance educational outcomes. For instance, adaptive learning strategies that cater to individual modalities, such as employing diagrams for visual learners or discussion-based approaches for verbal learners, have been shown to be effective (Swanson and Hoskyn, 2001). Similarly, learners with robust working memory capacities tend to excel in multitasking and complex problem-solving, whereas those with deficits benefit from structured and sequential methods (Gathercole et al., 2008). In addition, differences in processing speed suggest that traditional time-constrained assessments may disadvantage some students, highlighting the need for flexible pacing and differentiated instruction (Kail and Hall, 1994). Moreover, when learners are aware of their own cognitive strengths and limitations, they can leverage metacognitive strategies to improve self-regulated learning and academic performance (Zimmerman and Schunk, 2011). Finally, recognizing and addressing individual cognitive profiles enables the development of personalized teaching strategies that optimize learning experiences, thereby fostering academic success and increased self-efficacy (Orsoni et al., 2023). Clustering constitutes a pivotal domain within unsupervised pattern recognition and can be a relevant approach in finding suitable students cognitive profile (Orsoni et al., 2023). Its fundamental purpose is to partition a collection of unlabeled samples into distinct subsets according to a predefined objective function. The primary aim is to minimize inter-partition similarity while concurrently enhancing intra-partition similarity (Jayanth Krishnan and Mitra, 2022). However, the effectiveness of clustering solutions depends on multiple factors, including data characteristics and algorithm selection. Recently, confirmatory factor analysis (CFA) combined with latent profile analysis (LPA) (Schuster and Krogh, 2021) and factor mixture modeling (FMM) (Lubke and Muthén, 2005) has emerged as a robust approach for classifying learners' cognitive profiles by identifying underlying cognitive constructs and grouping individuals into latent classes based on their performance data. This methodology has been applied in various domains, including gifted education (Mammadov et al., 2016), in the analysis of self-regulated learning strategies in university students (Ning and Downing, 2014), and in the identification of preclinical cognitive phenotypes for Alzheimer's disease (Hayden et al., 2014). FMM, by following this line, merges continuous factor structures with categorical latent class distinctions. A work of Gomez and Vance (2014) presented the application of this method in the classification of cognitive heterogeneity in the co-occurrence of the childhood syndromes. Overall, the integration of CFA, LPA, and FMM facilitates a nuanced understanding of cognitive heterogeneity, thereby informing the development of personalized educational and psychological interventions. However, recent advances in deep unsupervised learning have significantly advanced latent class analysis, with methods such as autoencoders (AEs) and variational autoencoders (VAEs) proving particularly effective at enhancing clustering performance. These techniques encode high-dimensional data into compact, interpretable latent spaces that clarify intrinsic group structures (Lin et al., 2020; Yan et al., 2023). For instance, Eskandarnia et al. (2022) demonstrated the utility of combining dimensionality reduction with clustering algorithms for smart meter load profiling, while Peng et al. (2018) employed a structured autoencoder framework to map data into non-linear latent spaces optimized for subspace clustering. Further refinements to these architectures include Jiang et al. (2017), who introduced variational deep embedding (VaDE), integrating VAE with Gaussian mixture models to improve cluster separability. Subsequent innovations in VAE-based models, such as Kopf et al. (2021) mixture-of-experts (MoE), and Sarkar et al. (2020) Gaussian Mixture Variational Autoencoder and hybrid Conditional GMVAE for game design applications, have expanded the methodological toolkit for domain-specific clustering challenges.

This study compares factor mixture modeling (FMM) and Conditional Gaussian Mixture Variational Autoencoder (CGMVAE) as methods for analyzing cognitive profiles derived from six cognitive dimensions across multiple age groups. Notably, this research presents the first application of the CGMVAE architecture within the domain of cognitive profile analysis. Our core objectives are to (1) evaluate how effectively FMM and CGMVAE identify underlying latent cognitive structures, (2) characterize the distinct cognitive profiles each method reveals, and (3) analyze age-related trends in the distribution of these profiles.

## 2 Methods

### 2.1 Participants and instrument

All procedures adhered to the ethical standards established by national committees on human experimentation and were in accordance with the Helsinki Declaration of 1975, as revised in 2008. Approval for the study was obtained from the University of Bologna Bioethics Committee. Both parents and youths provided written and online informed consent to participate in the study.

**Figure 1:**
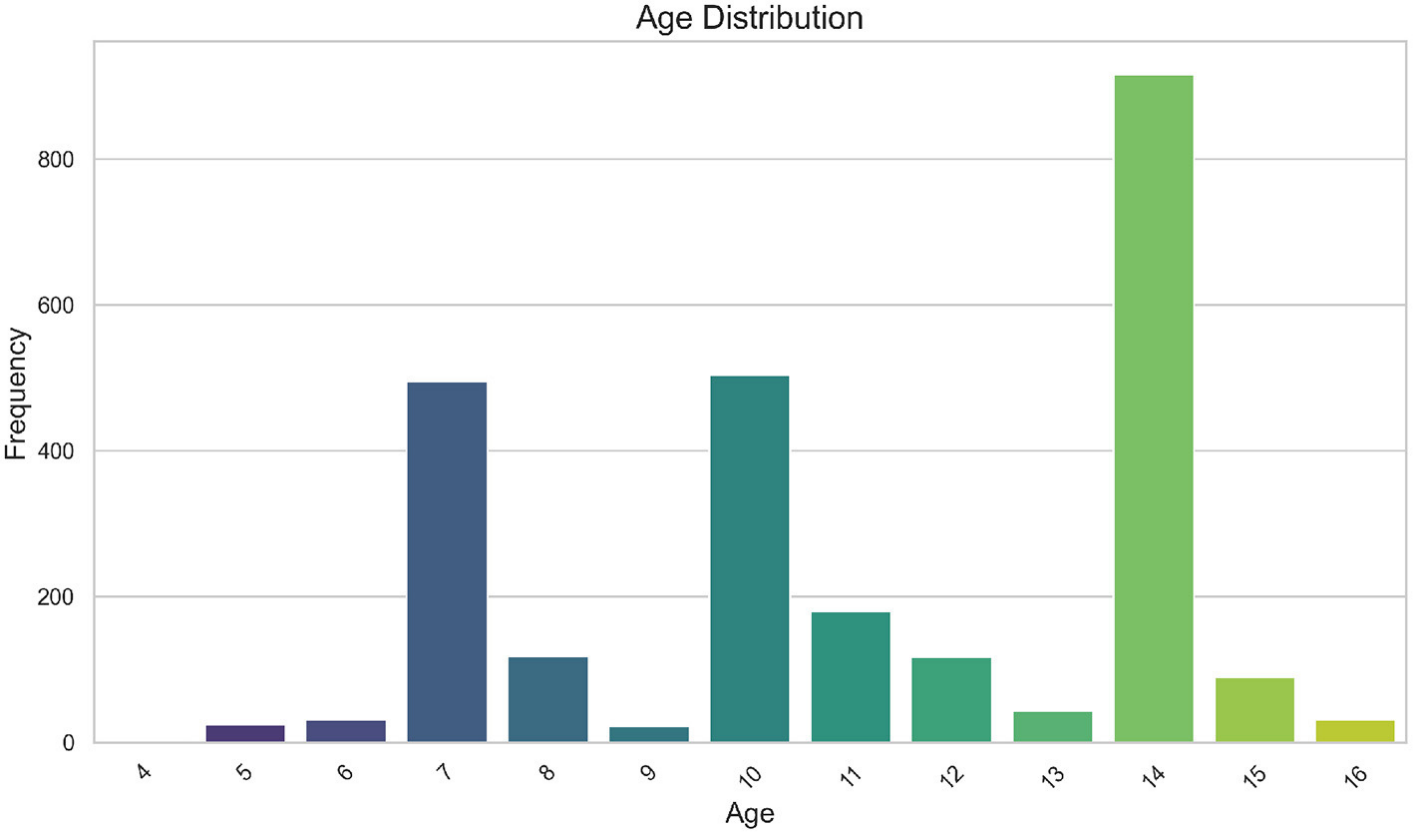
Bar chart of the sample age distribution.

A total of *n* = 2570 participants with an age range between 4 and 16 years old were considered eligible for subsequent analyses. Figure 1 displays the distribution of sample ages. Due to the presence of a trimodal pattern in age variable, both the FMM and CGMVAE methodologies employed a binned approach for the conditioned variable. Participant ages were then grouped into three categories: [0–8), [8–12), and [12–100), containing *n* = 670, *n* = 821, and *n* = 1, 079 samples, respectively.

The cognitive abilities of all paticipants were evaluated using an online digital game called PROFFILO, specifically designed for assessing students' cognitive profiles (Orsoni et al., 2023). The instrument convergent validity and specifications were already included in other published studies (Orsoni et al., 2021, 2023). In brief, the PROFFILO assessment comprised six distinct sub-tests (games), each tailored to evaluate a specific cognitive function, namely, logical reasoning, visuospatial attention, motion perception, phonological awareness, verbal comprehension, and working memory, and lasted 20–25 min per participant. A total of 54 items have been administered to all the participants divided as follows:

- **Logical reasoning:** 15 items. The test consists of a series of visual pattern matrices, each with one missing part. The task for the test taker is to identify the missing piece from multiple choices. The data are binary, representing 0 for incorrect and 1 for correct answers.
- **Visuospatial attention:** 3 items. The task requires the individual to focus their attention to specific visual elements in space, by responding to specific cues while ignoring distractions. The data is continuous in a range between 0 and 1.
- **Motion perception:** 5 items. In the current task, participants have to recognize directions of moving stimuli, obtained from white dots moving against a black background. This task allows to assess the subject's motion perception skills. The data are binary, representing 0 for incorrect and 1 for correct answers.
- **Phonological awareness:** 13 items. In the tasks, the test taker is presented with two auditory stimuli, and the task requires selecting the word that corresponds to a word that actually exist, while disregarding the non-word counterpart. The data are binary, representing 0 for incorrect and 1 for correct answers.
- **Verbal comprehension:** 17 items. The test involves the presentation of spoken sentences or phrases to the test taker, who is then required to select a corresponding picture that accurately represents the meaning of the presented linguistic content. The data are binary, representing 0 for incorrect and 1 for correct answers.
- **Working memory:** 1 item. The test involves presenting a participant with a sequence of numbers and then asking them to recall the items in reverse order. The length of the sequence increases proportionally with the participant's performance improvement. The test is interrupted after two consecutive errors. The data is continuous, ranging from a minimum value of 0.

### 2.2 Factor mixture modeling

Following the seminal work by Lubke and Muthén (2005), factor mixture modeling (FMM) combines factor analysis and latent class analysis to identify latent cognitive structures and classify individuals into distinct subgroups. In this study, we employed a FMM approach to investigate the underlying structure of the students cogntive profiles. By following the work of Clark et al. (2013), two FMM specifications were estimated: FMM-1 and FMM-2. In FMM-1, or the latent class factor analytic (LCFA) model, the item thresholds and factor loadings were constrained to be invariant across latent classes, with the only class-specific parameter being the factor mean α. In this specification, the factor covariance matrix Ψ was fixed at zero, thereby assuming no within-class heterogeneity beyond differences in factor location.

By contrast, FMM-2, or mixture factor analysis, allowed for a more flexible representation of the latent structure. Although it maintained invariant item thresholds and factor loadings as in FMM-1, instead of setting the factor variances and covariances to zero, they are now freely estimated in each of the classes. This modification enabled the model to capture also non-normal distributions of the latent variable, thus accommodating variability both between and within diagnostic categories.

As discussed in Clark et al. (2013), we adopted a systematic, multi-step approach to develop the FMM-1 and FFM-2, drawing from latent class analysis (LCA) and factor analysis (FA) as foundational paradigms. Initially, LCA models with an increasing number of classes and FA models with an increasing number of factors were fitted. This dual fitting strategy provided baseline solutions against which the more complex FMM could be compared, thereby informing decisions about model complexity and indicating when further increases in the number of classes or factors might be unnecessary. The next phase involved fitting a simple FMM with two latent classes and one latent factor. Subsequent models were estimated by gradually increasing the number of classes. This step allowed for an initial exploration of how class heterogeneity is captured in the presence of a single factor and served as a starting point for progressively building model complexity. Building on the simple FMM, the number of latent factors was increased from one to two, and the number of classes was again systematically increased in parallel. This iterative process, first varying the number of classes, then the number of factors, aimed to identify the point at which the model best captured the underlying structure of the data. The same pattern of complexity escalation was applied for both FMM-1 and FMM-2 models. To determine when to stop increasing the number of classes and factors was guided by the optimal number of classes from the LCA and the optimal number of factors from the FA, ensuring a parsimonious yet explanatory solution. After exploring different combinations of classes and factors, the best-fitting FMM was selected based on Bayesian information criteria (BIC), and Akaike information criterion (AIC) indices. Finally, the best FMM was compared with the optimal LCA and FA models. This comparison was essential to ensure that the integrated FMM solution provided superior fit and a more parsimonious representation of the data relative to the simpler models.

Moreover, we incorporated the binned age covariate in the models. This approach, as outlined by Lubke and Muthén (2005), allowed us to model the effect on latent class membership via a multinomial logistic regression framework. Specifically, the covariate is used to predict class probabilities through regression parameters, which are updated during the M-step of the Expectation-Maximization (EM) algorithm. This approach enables us to account for observed heterogeneity by directly influencing latent class assignments while retaining the overall structure of the FMM.

### 2.3 Conditional gaussian mixture variational autoencoder

Variational autoencoders (VAEs) have emerged as a principled approach to learning deep latent-variable models, enabling flexible data representations in both generative and semi-supervised contexts (Kingma and Welling, 2014, 2019). Building on this foundation, we employ a Conditional Gaussian Mixture Variational Autoencoder (CGMVAE), which extends the standard VAE framework by integrating (1) a conditional variable (binned age in our case) alongside the primary input and (2) a Gaussian mixture prior to capture inherently multi-modal latent distributions (Jiang et al., 2017; Sarkar et al., 2020).

#### 2.3.1 Model architecture

**Figure 2:**
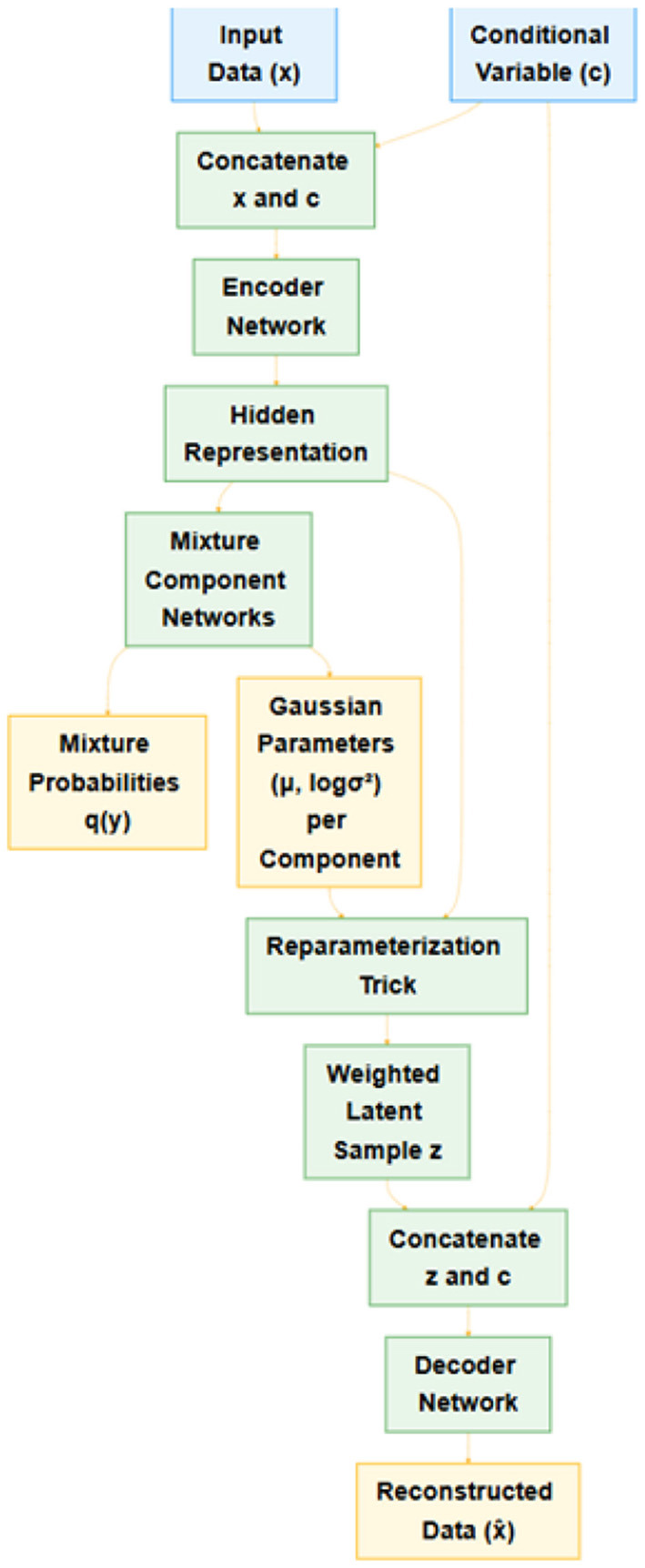
Graphical illustration of the Conditional Gaussian Mixture Variational Autoencoder. The input **x** is concatenated with condition **c** and then mapped to mixture probabilities and per-component Gaussian parameters. A latent sample **z** is drawn via the reparameterization trick and combined again with **c** for the reconstruction in the decoder.

Figure 2 provides an overview of the CGMVAE architecture. The encoder or recognition model maps the concatenation of the input vector **x** and the conditional variable **c** into hidden representations via a sequence of fully connected layers with non-linear activations. Specifically, the encoder outputs:

- **mixture probabilities** ***q***_*y*_, obtained through a softmax layer, representing a categorical distribution over *K* Gaussian components, and
- **component-wise Gaussian parameters**, t~he means ***μ***_*k*_ and log-variances ${\log\sigma }_{k}^{2}$ for each component *k*∈{1, …, *K*}.

Hence, each data point **x** is associated with a conditional distribution over **z**:


(1) $$\begin{array}{l} q\left(\text{z}|\text{x},\text{c}\right)=\overset{K}{\underset{k=1}{\sum }}q\left(y=k|\text{x},\text{c}\right)N\left(\mu_{k}\left(\text{x},\text{c}\right),\sigma_{k}^{2}\left(\text{x},\text{c}\right)\text{I}\right). \end{array}$$

Using the reparameterization trick, we draw latent samples **z** from each component in a differentiable manner (Kingma et al., 2015). The final latent representation is computed as the weighted sum of samples across all *K* components, weighted by *q*(*y* = *k*∣**x**, **c**).

The **decoder** or generative model receives the latent sample **z** (combined with the same conditional variable **c**) to reconstruct the original input **x**. In particular, **z** and **c** are concatenated and passed through a symmetric stack of fully connected layers, culminating in a final output layer with either:

- a Sigmoid for binary and normalized data or
- a linear output for continuous-valued features.

The decoder thus models $p\left(\text{x}|\text{z},\text{c}\right)=N\left(\hat{\text{x}},\sigma^{2}\text{I}\right)$ for the continuous case or a Bernoulli-based parameterization for the binary/normalized data case (Kingma and Welling, 2019).

#### 2.3.2 Loss function and training

The total objective is to minimize the negative evidence lower bound (ELBO), augmented to handle the mixture and conditional terms:


(2) $$ℒ(x,c)\;=\;\underset{\text{Reconstruction Loss}}{\underset{︸}{E_{q(z|x,c)}[-\log p(x|z,c)]}}\;+\;\beta \;\underset{\text{Regularization Term}}{\underset{︸}{D_{\text{KL}}[q(z|x,c)\|p(z|c)]}},$$

where the KL divergence *D*_KL_ is computed between the encoding distribution *q*(**z**|**x**, **c**) and the mixture prior *p*(**z**|**c**), itself represented by learnable ***μ***_prior_, for each mixture component. The scalar β∈(0, 1] is a hyperparameter that balances the KL term and the reconstruction accuracy regulating the emphasis placed on disentangled latent representations and subpopulation discovery (Higgins et al., 2016).

The reconstruction loss is implemented as the minimization of the mean squared error (MSE). Each training iteration thus optimizes:


(3) $$\begin{array}{l} \text{Total Loss}=\text{Reconstruction Loss}+\beta D_{\text{KL}}, \end{array}$$

where the gradient of the loss is backpropagated through the latent sampling by means of the reparameterization trick (Kingma et al., 2015):


(4) $$\begin{array}{l} \text{z}=\mu_{z}+\sigma_{z}⊙\epsilon ,\;\epsilon \sim N\left(0,\text{I}\right). \end{array}$$

#### 2.3.3 Implementation details

We developed the CGMVAE in PyTorch. The encoder combines the original input (**x**) and condition vector (**c**), passing them through fully connected layers (each comprising linear transformations, ReLU activations, and batch normalization). The final shared layer outputs (i) logit scores for the mixture probabilities **q**_*y*_, (ii) ***μ***, and (iii) **logσ**^2^ for each of the *K* mixtures, reshaped for convenience as (batch_size, n_components, latent_dim). The decoder mirrors this structure by mapping (sampled) latent variables back to input space, producing the reconstructed $\hat{\text{x}}$.

A notable extension of our CGMVAE is the mu_prior and logvar_prior parameters, which are learned for each mixture component, providing a flexible Gaussian mixture prior that can adapt to complex, multi-modal latent structures in real-world data, a relevant aspect due to the nature of datasets that often have subpopulation heterogeneity.

#### 2.3.4 Training pipeline and hyperparameter optimization

We trained the CGMVAE using:

- **Data loaders:** Mini-batched training and validation sets.
- **Optimization:** Adam is configured via a learning rate, weight decay, and an optional gradient clipping that prevent the gradients from excessively large values.
- **Scheduler:** A ReduceLROnPlateau to automatically adjust the learning rate upon plateauing validation losses.
- **Checkpointing:** Periodic saving of model weights and optimizer states.

Validation performance is monitored by computing the reconstruction and KL terms on a held-out set, and the best model weights are saved based on minimal validation loss. In addition, we implemented a hyperparameter optimization routine leveraging Optuna framework to systematically explore a search space for latent dimensions, number of mixture components, learning rate, and free bits parameter. This latter parameter helps to prevent the model from over-regularizing the latent space by ensuring that each latent variable contributes at least a minimal amount of information. The final model is retrained on the full training set using the best hyperparameters and stored for subsequent analyses.

### 2.4 Feature focused interpretation of cluster solution and cluster quality assessment

The cluster interpretation was performed using a feature-focused analysis approach on the trained CGMVAE model and the winner FMM solution. For each cluster, we performed statistical analyses including univariate feature analysis computing means, standard deviations, along with normalized differences from global statistics using z-scores.

Cluster quality has been assessed on both methods by computing standard and fuzzy cluster quality indices on the latent space representations. In the CGMVAE solution, we derived the latent features via the model's encoder and determined hard assignments on the fuzzy membership matrix. The Silhouette Score (Rousseeuw, 1987) (which measures the cohesion and separation of clusters), the Calinski-Harabasz Index (Calinski and Harabasz, 1974) (which quantifies the ratio of between-cluster dispersion to within-cluster dispersion), and the Davies-Bouldin Score (Davies and Bouldin, 1979) (which assesses average cluster similarity, with lower values reflecting better separation) were then estimated in both CGMVAE and FMM solution. In addition to these, we computed fuzzy clustering metrics to capture the uncertainty in cluster membership for CGMVAE. The Xie-Beni Index (XB) (Xie and Beni, 1991) provides an assessment of the compactness and separation of fuzzy clusters. The fuzzy partition coefficient (FPC) (Wu and Yang, 2005), which averages the squared membership degrees over all samples, indicates the crispness of the clustering, while the partition entropy (PE) (Bezdek, 1981) quantifies the fuzziness of the assignment by measuring the entropy of the fuzzy membership values.

### 2.5 Software and packages

The analyses were conducted using a system equipped with an NVIDIA GeForce RTX 2060 graphics card and an Intel i7-10750H CPU operating at 2.60GHz. Python v3.9.16 (Van Rossum and Drake, 2009) was used for the analyses. The FMM and CGMVAE were performed on Python by using the *scikit-learn* library (Pedregosa et al., 2011). The *Pytorch* library v.2.0.0 (Paszke et al., 2019) was used for CGMVAE model computation, *Optuna* for hyperparameter optimization (Akiba et al., 2019), and *Weight & Biases* for experiment tracking (Biewald, 2020).

## 3 Results

### 3.1 Factor mixture modeling

The investigation of the underlying structure of students' cognitive profiles by using factor mixture modeling (FMM), comparing specifications with class-invariant (FMM-1) and class-specific (FMM-2) factor structures, followed the approaches outlined by Lubke and Muthén (2005) and Clark et al. (2013). Prior to fitting FMMs, baseline LCA and FA models were estimated to guide the FMM specification process. The best-fitting FA model yielded a BIC of 110,749.98 with five latent factors, while the best LCA model resulted in a BIC of 117,211.73 with two latent classes. Based on these analyses and theoretical considerations, subsequent FMM analyses explored models with up to two latent classes and five latent factors. As outlined in the Methods section, we systematically fitted FMM-1 and FMM-2 models, incorporating the binned age covariate to predict latent class membership. The complexity of the models was increased incrementally, starting with two classes and one factor for both the models.

Based on the BIC values showed in Table 1, the FMM-1 model with 2 classes and 1 factor provided the most parsimonious fit to the data (BIC = 94,285.72). This model outperformed the best-fitting FMM-2 model (BIC = 94,439.41 for the 2-class, 1-factor solution) and was considerably better than the optimal baseline LCA (BIC = 117,211.73) and FA (BIC = 110,749.98) models. The selected FMM-1 model identified two latent classes with distinct profiles characterized by different means on a single underlying cognitive factor. The estimated class proportions were 29.14% for Class 1 and 70.86% for Class 2.

**Table 1:** Model comparison between FMM-1 and FMM-2 models.

**Model specification**	**Factors**	**BIC**	**AIC**
FMM-1	1	**94,285.72**	**93,519.15**
		2	96,140.41	95,046.15
		3	100,594.34	99,172.39
		4	108,745.80	106,996.15
		5	102,263.78	100,186.44
FMM-2	1	94,439.41	93,661.14
		2	95,124.53	93,995.16
		3	96,447.74	94,955.57
		4	97,587.32	95,720.64
		5	98,186.11	95,933.22
Best factor analysis (FA)		110,749.98	–
Best latent class analysis (LCA)		117,211.73	–

BIC, Bayesian information criterion; AIC, Akaike information criterion. Lower values indicate better model fit relative to complexity. The best-fitting model based on BIC is shown in bold. BIC values for the best comparison FA and LCA models are provided.

**Figure 3:**
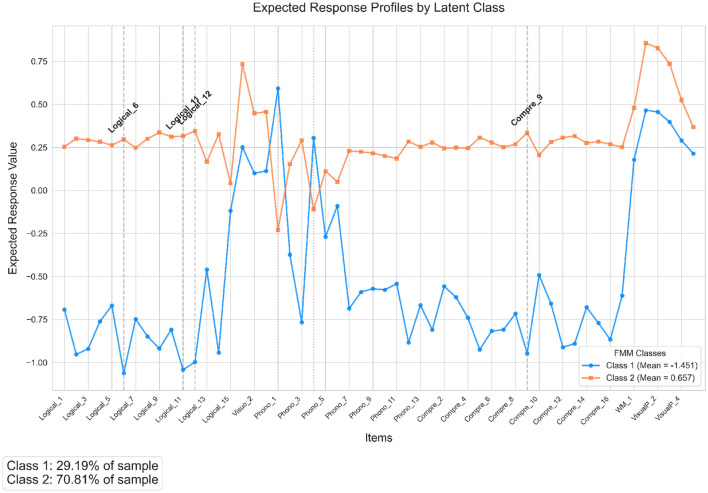
Expected response profiles for the two latent classes derived from the Factor Mixture Model (FMM). The blue line (circles) represents Class 1 (factor mean ≈ −1.45), and the orange line (squares) represents Class 2 (factor mean ≈0.66). Profiles are calculated using class-specific factor loadings and the respective class factor means, illustrating how expected item responses vary. Items exhibiting the largest absolute differences between classes are highlighted with gray dashed vertical lines and labels. Items with negative factor loadings (based on Class 1 loadings, indicated by red dotted vertical lines) show reversed patterns where Class 1 has higher expected responses.

The selected best-fitting model posits that the underlying cognitive factor structure (defined by item intercepts and factor loadings) is invariant across classes. Differences between the classes arise solely from variations in the mean of the single latent factor. The estimated factor means for the two classes were Class 1 Mean (α_1_): -1.451 and Class 2 Mean (α_2_): 0.657. These means present a clear separation between the classes on the latent cognitive dimension captured by the factor. Class 1 exhibits a significantly lower mean factor score compared to Class 2. As shown in Figure 3, Class 1 represents a subgroup generally expected to perform lower across the assessed cognitive tasks, while Class 2 represents a subgroup expected to perform higher. The expected response profile for each class on each item can be derived using the estimated item intercepts, the invariant factor loadings, and the class-specific factor means. The magnitude of the difference between the two classes on any given item is proportional to the magnitude of that item's factor loading; items with stronger positive loadings (e.g., Logical_6 [loading=0.73], Logical_11 [0.72], Logical_12 [0.69], Phonological Comprehension_9 [0.65]) show the largest expected performance gap favoring Class 2. Conversely, for items with negative loadings, this pattern is reversed. Based on the invariant loadings, the items 1 and 4 of Phonological task showed loadings equal to −0.41, and −0.21, respectively, are expected to show higher average responses for Class 1 compared to Class 2.

By focusing on the covariance role of binned age variable, the analysis revealed a significant association between age and the probability of belonging to Class 1 relative to Class 2. The estimated regression coefficient for Age_C predicting membership in Class 1 versus Class 2 was equal to −0.963. This negative coefficient indicates that as the binned age variable increases, the log-odds of being classified into Class 1 (the lower-performing profile) compared to Class 2 decrease. Exponentiating the coefficient yields an odds ratio (OR) of approximately 0.382. This suggests that for each one-unit increase in the binned age variable, the odds of an individual belonging to Class 1 rather than Class 2 are multiplied by 0.382, representing a decrease of approximately 61.8%. Therefore, higher values on the covariate are strongly associated with a reduced likelihood of membership in the lower-performing Class 1 relative to the higher-performing Class 2. The intercept term for the comparison of Class 1 versus Class 2 was −0.048, corresponding to an odds ratio of approximately 0.953. This suggests that when the binned age is at its mean (value of 0), the baseline odds of being in Class 1 vs. Class 2 are nearly the same, although slightly lower for Class 1.

### 3.2 CGMVAE

**Figure 4:**
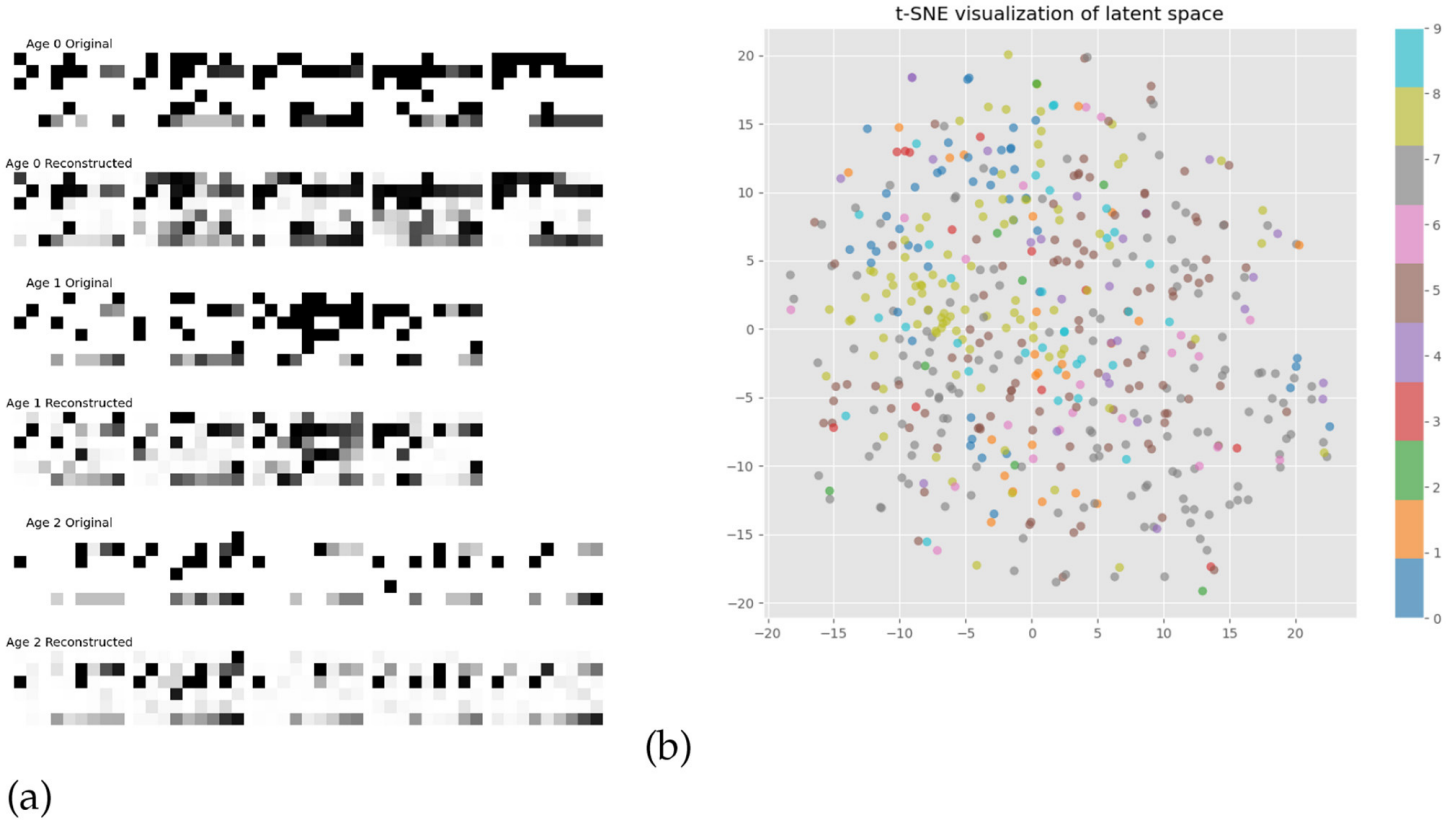
Model performance evaluation: **(a)** Reconstruction errors across binned age groups and **(b)** t-SNE projection of latent representations, with colors denoting the 10 Gaussian mixture components.

The CGMVAE architecture achieved an optimal performance after 15 epochs with a validation loss equal to 1.516. The encoder architecture comprised a fully connected layer followed by ReLU activation and batch normalization, branching into separate networks for computing mixture components *q*(*y*|*x*), means (μ), and log-variances (logσ^2^). The decoder reconstructed the input features through a mirror architecture, maintaining the same hidden dimension while incorporating the conditional information. The winner parameters showed an encoder hidden layer of 201 units feeding into a latent space of 58 dimensions, structured as a mixture of 10 Gaussian components. Training employed the Adam optimizer with a learning rate of 9.68 × 10^−4^ and a weight decay of 4.00 × 10^−5^, with the variational objective tuned via a β of 0.051 and a free bits threshold of 0.048. Gradient clipping at 0.644 further ensured stable parameter updates during training. Figures 4a, b showed graphically the reconstruction error by binned ages and the latent space of the two dimensions by applying the t-SNE visualization analysis (van der Maaten and Hinton, 2008). As depicted in Figure 4a, the reconstruction error analysis demonstrates the model's capability to preserve essential data features across all age bins, with reconstructed samples closely matching their original counterparts. However, subtle variations in reconstruction quality are observed across the age spectrum, suggesting age-specific patterns in data complexity and representational characteristics.

### 3.3 Cluster characteristics by ages and features

**Figure 5:**
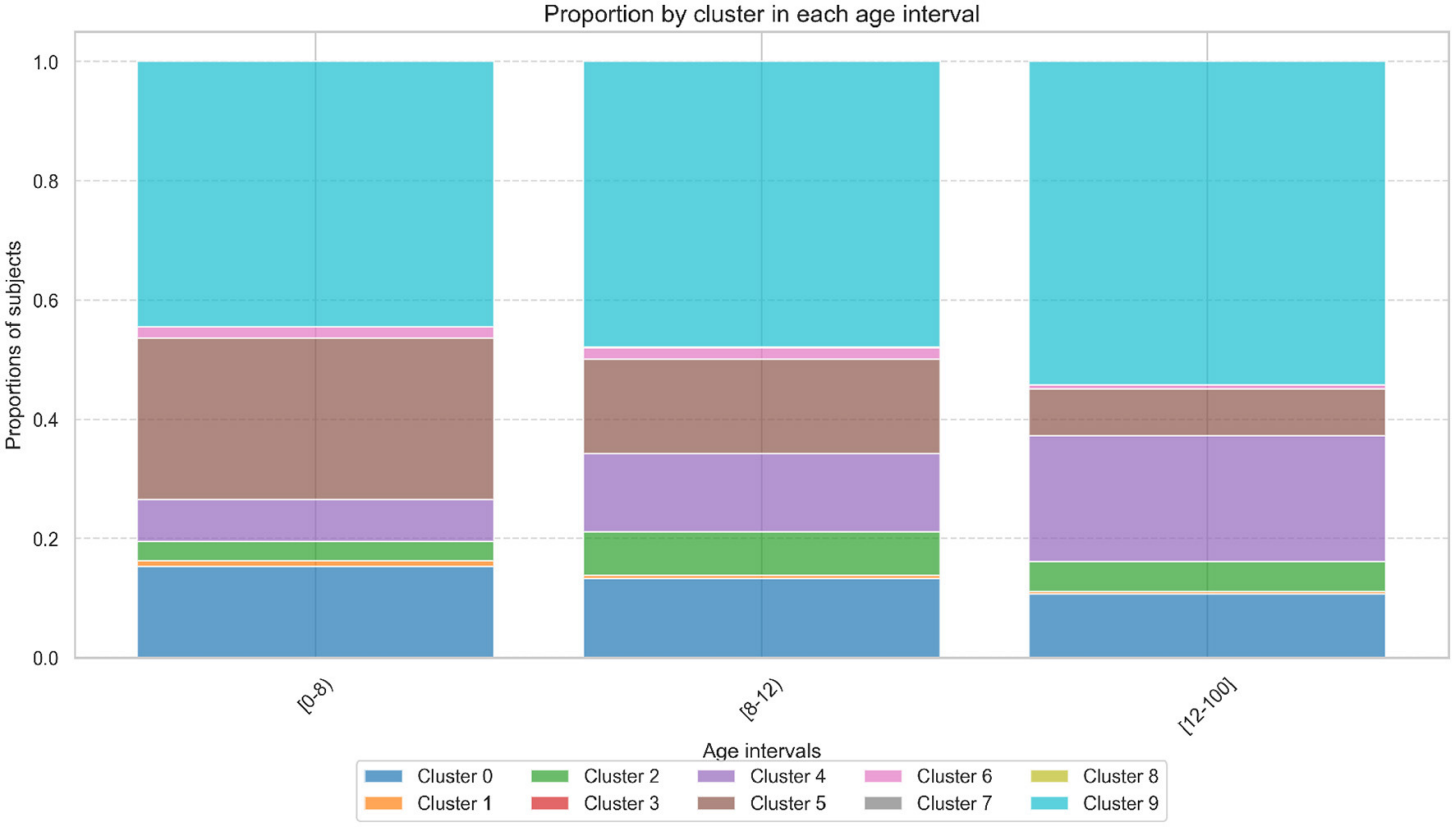
Proportion of samples within each cluster across binned age intervals. The bar plot highlights the representation of age groups [0–8), [8–12), and 12+ within the cluster solution.

Figure 5 illustrates the distribution of binned age groups across the proposed cluster solution, where Cluster 9 emerges as the most representative across all age intervals, with its representativeness progressively increasing from 44% in the [0–8) age group to 48% in [8–12) and peaking at 54% for individuals older than 12 years.

In contrast, the proportion of subjects in Cluster 5 decline with age, starting at 27% in the [0–8) age group, dropping to 16% in [8–12), and further decreasing to 8% in the [12–100] age group. Meanwhile, Cluster 0 shows an almost homogeneous distribution across the age groups with proportions of 15%, 13%, and 11%, respectively, and Cluster 4 exhibits an increasing trend with age, from 7% in [0–8) to 13% in [8–12) and 21% in [12–100]. All remaining clusters have a homogeneous representation and are less than 10% across all age ranges.

We focused our detailed analysis on clusters with statistically meaningful sample sizes (*n* ≥ 10 subjects). Following this criterion, Clusters 3, 7, and 8 were excluded from further investigation due to insufficient sample representation. Complete z-scores and detailed cluster characteristics for all features are provided in Appendix A.

**Cluster 9**. It is the most representative of the entire sample. It exhibits a balanced cognitive profile with performances classified as average across various domains. Among these, motion perception and logical reasoning demonstrated subtle but consistent strengths, with slight positive deviations in verbal and phonological abilities. By investigating z-scores, motion perception showed minor enhancements across tasks, with the strongest observed in tasks 5 (Z = +0.24) and 3 (Z = +0.23) of the test. Logical reasoning tasks followed a similar trend, with task 1 (Z = +0.21) performing the best. Verbal comprehension (e.g., task 17, Z = +0.20), phonological awareness (task 5, Z = +0.16), and working memory (Z = +0.04) also showed slight improvements, albeit within the average performance range. Overall, the cluster reflects a cognitively stable and homogeneous group, with average performance across tasks and minor variability in specific domains, such as phonological awareness, which included only a slight underperformance in task 8 (Z = –0.13).

**Cluster 5**. It demonstrates a distinctive cognitive pattern characterized by pronounced strengths in visuospatial attention (task 3: Z = +0.45), above average logical reasoning abilities (e.g., task 15: Z = +0.34; task 11: Z = +0.29), and working memory processing (Z = +0.18), while exhibiting some weaknesses in phonological processing (task 6: Z = –0.52; task 4: Z = –0.48). The cluster's profile is further distinguished by average motion perception capabilities and verbal comprehension.

**Cluster 4**. It exhibits a cognitive profile characterized by below-average performance in perceptual-motor domains, with consistent reductions across all motion perception variables (ranging from Z = -0.51 to Z = -0.36) and mild impairments in logical reasoning, accompanied by modest difficulties in visuospatial information integration, and in several verbal comprehension subdimensions (e.g., tasks 8, 15, 2, and 9, with reductions ranging from Z = –0.39 to Z = –0.19). However, strengths emerge in the phonological domain, particularly evidenced in task 4 (Z = +0.41), task 5 (Z = +0.18), and task 9 (Z = +0.11).

**Cluster 2**. It presents a distinctive cognitive pattern characterized by average global performance with specific vulnerabilities and strengths across different domains. In the domains of phonological awareness and verbal comprehension, the subjects have average performances in mostly all domains with few exceptions of vulnerabilities (e.g., phonological awareness, task 5: Z = –0.84; verbal comprehension, task 3: Z = –0.72; task 11: Z = –0.64). However, these limitations are counterbalanced by strengths in logical reasoning tasks (task 5: Z = +0.39; task 13: Z = +0.29) and motion perception (task 2: Z = +0.34; task 1: Z = +0.27 SD). The working memory ability is slight below average (Z = –0.22). This cluster indicates a cognitive profile characterized by local specialization rather than integrated processing. The cluster's performance suggests compensatory mechanisms where visual-perceptual and logical abilities potentially offset weaknesses in phonological and verbal domains, pointing to alternative cognitive processing strategies.

**Cluster 0**. It exhibits a distinctive cognitive profile characterized by slight but systematic reductions across several domains, with the most marked deficits observed in verbal comprehension, logical reasoning, and motion perception. Specifically, verbal comprehension tasks such as task 17 show a significant drop (Z = −0.62), while logical reasoning measures and motion perception variables consistently fall below the global average. Visuospatial attention is also modestly affected, with reductions approximately −0.28 to −0.33 in the z-scores, whereas phonological awareness remains comparatively preserved. Working memory showed a slight below performance (Z = −0.12). Overall, the profile of Cluster 0 reflects uniform mild reductions in cognitive performance, particularly in logical and perceptual-motor tasks, with potential localized compensations in verbal and phonological processing.

**Cluster 1**. With a small sample size (*n* = 17), it demonstrates a highly distinctive cognitive profile characterized by severe deficits coupled with isolated compensatory mechanisms. The most pronounced impairments are observed in verbal comprehension (task 3: Z 0 −2.05), logical reasoning (task 9: Z = −1.09), visuospatial attention (task 1 and 2: Z = −0.59 and −0.41, respectively), and slight in working memory (Z = −0.37). However, the cluster exhibits remarkable compensatory strengths in phonological awareness (task 4: Z = +0.70) and motion perception (task 2 and 3: Z = +0.33 and 0.30). This profile suggests a severe deficits in core verbal and logical domains potentially driving compensatory developments in phonological and perceptual processing. The small sample size warrants careful interpretation, but the consistent pattern of extreme polarization across multiple measures suggests a distinct neurocognitive phenotype.

**Cluster 6**. With a small sample size (*n* = 37) exhibits a highly atypical cognitive profile characterized by severe deficits across multiple domains, particularly in verbal comprehension (task 17: Z = −1.42), logical reasoning (task 9: Z = −1.06), and phonological awareness (task 13: Z = −0.99). Working memory shows moderate impairment (Z = −0.46), potentially contributing to broader cognitive difficulties. However, the cluster demonstrates remarkable isolated strengths in motion perception (task 4: Z = +0.55; task 3: Z = +0.37). The profile aligns with potential neurodevelopmental conditions characterized by significant language and executive function impairments alongside isolated areas of preserved or enhanced abilities.

### 3.4 Comparing FMM and CGMVAE cluster quality

The cluster quality showed very different results in the two proposed solutions. By focusing on FMM, the solution in the model's latent factor space demonstrates exceptionally good separation between classes based on the three metrics. First, the Silhouette score of 0.959 is very close to the maximum possible value of 1.0, indicating that observations within each cluster are tightly grouped while remaining well separated from other clusters.

Next, the Davies-Bouldin Index registers at an extremely low of 0.074, reinforcing the notion that clusters are highly distinct; as this index approaches zero, the average distance between clusters grows relative to their internal dispersion. Finally, the Calinski-Harabasz Index reaches 113,432.648, a notably high value that further confirms well-defined cluster structure. Together, these metrics lend strong support to the conclusion that the Factor Mixture Model has partitioned the data into clear latent classes in the factor space. Conversely, the solution proposed by the CGMVAE algorithm indicates that the clusters are relatively weak overall. A negative silhouette score (–0.008) and low Calinski-Harabasz index (7.005), together with high Davies-Bouldin (4.244) and Xie-Beni (32.783) indices, showed that the clusters are not highly distinct and that there is considerable overlap between them. In addition, the fuzzy partition coefficient value (0.109) and the high partition entropy (2.257) suggest that membership probabilities are large, which aligns with the need for cautious interpretation of clusters with small sample sizes (e.g., Clusters 1 and 6). Then, even though the detailed profiles and age distributions offer rich insights into cognitive heterogeneity, the overall clustering solution appears to be less sharply delineated, pointing out that the differences, while present, may reflect subtle gradations rather than clear separations.

## 4 Discussion

This study presents a comparative analysis between a factor mixture modeling (FMM) and a deep clustering based approach, a Conditional Gaussian Mixture Variational Autoencoder (CGMVAE) in cognitive profile analysis. These two methods have been evaluated in their effectiveness across six cognitive dimensions assessed through the PROFFILO game (Orsoni et al., 2021) and multiple age groups. Our results indicate that the FMM, particularly the simpler two-class, one-factor solution (FMM-1), produced exceptionally clear separations between classes, while the CGMVAE uncovered more nuanced but less well-delineated cluster structures.

From a model-fitting perspective, the FMM excelled by identifying two broadly distinct latent classes. The solution's robustness was evidenced by near-ideal clustering metrics. Its Silhouette Score (0.959) approached the upper limit of 1.0, signifying minimal overlap between classes and tight intra-class homogeneity. Similarly, a low Davies-Bouldin Index (0.074) and high Calinski-Harabasz Index provided strong convergent evidence for well-defined class boundaries. This clarity suggests that the single latent factor effectively captured most of the individual differences in our data. Class membership probabilities similarly showed clear differences, implying that each subgroup reflected a meaningful cognitive profile, one with lower overall performance and one with higher overall performance on the six different cognitive tasks. The covariance effect showed an effect of binned age. Higher ages are strongly associated with a reduced likelihood of membership in the lower performing Class 1 relative to the higher-performing Class 2.

By contrast, the CGMVAE, which is designed to handle complex, non-linear relationships, identified multiple clusters with overlapping features and fuzzy boundaries. While this suggests deeper insight into potentially subtle cognitive differences, cluster quality metrics pointed to weaker overall separability. Negative Silhouette Scores (–0.008) underscore difficulty in distinguishing unique cluster identities; a high Davies-Bouldin Index (4.244) and small Calinski-Harabasz Index (7.005) further reveal limited divergence across the identified clusters. In addition, the fuzzy partition coefficient (0.109) and elevated partition entropy (2.257) highlight considerable uncertainty in assigning individuals to specific clusters. These results implies that the CGMVAE captures more complex patterns but may be inappropriate when the research or intervention context demands a clear separation between groups. By investigating the reconstruction error analysis, it showed robust performance across all age bins, suggesting effective preservation of essential cognitive characteristics. The analysis of cluster distributions revealed distinct developmental trajectories, with Cluster 9 emerging as predominant and showing increased representation with age (44% to 54%). Contrasting patterns were observed in Cluster 5 (declining from 27% to 8%) and Cluster 4 (increasing from 7% to 21%), while Cluster 0 remained relatively stable across age groups. By looking at the cluster characteristics, the analysis revealed distinct cognitive profiles that suggest different patterns of strengths, weaknesses, and potential compensatory mechanisms across neurocognitive domains. Cluster 9, representing the normative profile, displayed consistently average performance with subtle strengths in motion perception and logical reasoning (Z-scores approximately +0.20), serving as a baseline for comparison. In contrast, Clusters 1 and 6 exhibited the most atypical profiles, characterized by severe deficits in verbal comprehension (Z = –2.05 and –1.42, respectively) and logical reasoning, coupled with remarkable compensatory strengths in specific domains such as motion perception and phonological awareness. Three intermediate patterns emerged: Cluster 5 showed enhanced visuospatial attention (Z = +0.45) and logical reasoning abilities despite phonological processing weaknesses; Cluster 4 demonstrated consistent perceptual-motor deficits offset by phonological strengths; and Cluster 2 exhibited a profile of selective vulnerabilities in phonological and verbal domains counterbalanced by logical and motion perception strengths. Cluster 0 presented a unique pattern of mild but systematic reductions across multiple domains, particularly affecting verbal comprehension (Z = –0.62) and logical reasoning. These distinct profiles suggest different underlying neurocognitive profiles, potentially reflecting various developmental trajectories or compensatory mechanisms. The presence of both severe deficits and domain-specific strengths within the same clusters (particularly in Clusters 1 and 6) points to the possibility of neural reorganization and the development of alternative processing strategies.

In general, these results present a key methodological trade-off. FMM excels in parsimony and interpretability, generating a small number of highly distinct groups. This makes FMM particularly valuable for applications requiring clear categorical distinctions. Differently, the CGMVAE's flexible architecture can model richer interactions among variables, potentially unveiling hidden facets of cognitive variation that are not captured within simpler factor solutions. However, the cost is weaker cluster separation and greater membership ambiguity, which may complicate direct intervention or classification efforts.

## 5 Conclusion

This study compared factor mixture modeling (FMM) and Conditional Gaussian Mixture Variational Autoencoders (CGMVAE) for identifying cognitive profiles. The evidence showed that both methods offer unique advantages. The FMM provides clear, readily interpretable clusters, while CGMVAE reveals more complex, non-linear patterns in cognitive abilities.

The results highlighted how FMM analysis successfully identified two distinct and well-separated cognitive classes, with binned age significantly predicting class membership. This highlights FMM's strength in producing a parsimonious and statistically robust partition based on cognitive dimensions. Conversely, the CGMVAE uncovered multiple, more nuanced cognitive profiles characterized by specific combinations of strengths and weaknesses across domains. These profiles exhibited clear developmental trajectories linked to age. However, this increased granularity came at the cost of cluster quality; the CGMVAE clusters showed considerable overlap and lacked sharp boundaries, indicating weaker separation compared to the FMM solution. However, several limitations warrant consideration. Our FMM analysis did not extend to FMM-3 and FMM-4 models (Clark et al., 2013), which allow for class specific item parameters and could potentially capture more subtle forms of measurement non-invariance. The CGMVAE approach, despite its modeling power, presents practical hurdles. Its complexity (58 latent dimensions, 10 components) and high-dimensional latent space hinder interpretability and hidden the direct link between cognitive features and cluster assignments.

Furthermore, methodological constraints included binning age into broad categories due to a trimodal distribution, potentially masking finer developmental changes, and a lack of demographic data, limiting the examination of socioeconomic or cultural factors. The generalizability of the CGMVAE findings needs confirmation across different cognitive assessment tools as performance might be tool-specific. Finally, the significant computational resources and the inherent “black-box” nature of the CGMVAE could hinder its adoption and trust among practitioners in educational settings.

Future research should focus on validating these findings across diverse populations and assessment instruments. Integrating the strengths of both approaches, using FMM for initial broad classification and CGMVAE for subsequent refinement of subtle, non-linear variations within those classes, could offer a powerful hybrid strategy. Moreover, translating these complex analytical findings into actionable educational interventions remains a key objective.

In conclusion, FMM and CGMVAE serve different but complementary roles in cognitive profiling. FMM excels at providing clear, practical classifications, whereas CGMVAE offers a deeper lens into the subtle complexities and developmental dynamics of cognitive performance.

## Data Availability

The raw data supporting the conclusions of this article will be made available by the authors, without undue reservation.

## Appendix

### Clustering features characteristics

**Table 2:** z-Scores across cognitive domains and clusters.

**Cognitive domain**	**Cluster 0**	**Cluster 1**	**Cluster 2**	**Cluster 4**	**Cluster 5**	**Cluster 6**	**Cluster 9**
**Motion perception**							
Task 1	–0.51	+0.18	+0.27	–0.46	+0.21	+0.13	+0.17
Task 2	–0.49	+0.33	+0.34	–0.51	+0.10	+0.13	+0.20
Task 3	–0.53	+0.30	+0.20	–0.49	+0.05	+0.37	+0.23
Task 4	–0.29	+0.06	+0.12	–0.38	+0.02	+0.55	+0.16
Task 5	–0.37	+0.07	–0.01	–0.36	–0.11	+0.04	+0.24
**Logical reasoning**							
Task 1	–0.12	–0.28	–0.37	–0.37	–0.01	–0.81	–0.21
Task 2	–0.28	+0.08	+0.27	–0.13	+0.18	+0.30	+0.02
Task 3	–0.49	–0.40	+0.17	+0.11	+0.18	–0.34	+0.10
Task 4	–0.57	–0.10	+0.17	+0.00	–0.18	–0.26	+0.19
Task 5	–0.27	–0.86	+0.39	–0.43	+0.04	–0.01	+0.16
Task 6	–0.47	+0.27	+0.24	–0.08	+0.12	–0.48	+0.10
Task 7	+0.04	–0.13	+0.08	–0.25	+0.11	+0.01	+0.03
Task 8	–0.34	–0.02	+0.00	–0.34	+0.20	+0.01	+0.13
Task 9	–0.37	–1.09	+0.24	–0.10	+0.08	–1.06	+0.12
Task 10	+0.02	–0.24	–0.45	–0.36	+0.06	–0.61	+0.16
Task 11	–0.29	–0.33	+0.01	+0.07	+0.29	–0.42	–0.02
Task 12	–0.32	–0.10	+0.19	+0.03	+0.13	–0.19	+0.02
Task 13	–0.15	–0.31	+0.29	+0.15	+0.10	–0.25	–0.06
Task 14	–0.28	–0.86	–0.04	–0.07	+0.07	–0.77	+0.11
Task 15	–0.26	–0.13	+0.02	–0.12	+0.34	+0.09	–0.01
**Verbal comprehension**							
Task 1	+0.11	–1.01	+0.09	–0.11	+0.10	–0.56	+0.05
Task 2	–0.04	–0.91	–0.19	–0.21	+0.12	–0.56	+0.09
Task 3	–0.25	–2.05	–0.72	–0.05	+0.16	–0.86	+0.16
Task 4	+0.14	–0.60	–0.16	–0.02	+0.03	–0.12	–0.01
Task 5	+0.07	–0.81	+0.11	–0.22	+0.10	–0.60	+0.03
Task 6	–0.05	–0.29	+0.14	–0.02	+0.15	–0.48	–0.02
Task 7	+0.03	–0.32	+0.05	–0.04	+0.13	–0.40	–0.02
Task 8	+0.08	–0.50	+0.09	–0.39	+0.14	–0.67	+0.07
Task 9	+0.08	–0.25	+0.06	–0.19	+0.01	–0.39	+0.04
Task 10	–0.19	+0.44	+0.02	–0.06	–0.24	+0.08	+0.13
Task 11	+0.25	–0.33	–0.64	+0.02	+0.24	–0.44	–0.06
Task 12	–0.12	–0.11	+0.18	–0.07	+0.22	–0.08	–0.03
Task 13	–0.24	–0.29	+0.14	–0.00	+0.08	–0.24	+0.03
Task 14	+0.01	–0.49	–0.07	+0.02	+0.09	–0.34	–0.01
Task 15	–0.05	–0.23	+0.05	–0.24	+0.19	–0.55	+0.04
Task 16	+0.03	–0.32	+0.02	–0.12	+0.06	–0.40	+0.03
Task 17	–0.62	+0.01	–0.40	+0.01	+0.12	–1.42	+0.20
**Phonological awareness**							
Task 1	+0.09	+0.35	–0.03	–0.13	–0.16	+0.46	+0.05
Task 2	–0.05	+0.25	–0.03	+0.06	+0.06	–0.55	–0.01
Task 3	+0.03	+0.09	–0.10	+0.08	+0.04	+0.27	–0.04
Task 4	–0.10	+0.70	+0.04	+0.41	–0.48	+0.30	+0.03
Task 5	–0.23	–0.05	–0.84	+0.18	–0.19	–0.23	+0.16
Task 6	+0.12	–0.09	+0.05	–0.07	–0.52	+0.23	+0.14
Task 7	+0.11	–0.39	+0.11	+0.01	–0.02	+0.18	–0.04
Task 8	–0.01	+0.40	+0.35	+0.03	+0.25	+0.16	–0.13
Task 9	+0.08	+0.10	+0.20	+0.11	+0.16	–0.27	–0.12
Task 10	–0.00	–0.80	+0.11	+0.01	+0.04	–0.12	–0.01
Task 11	–0.12	+0.07	–0.15	–0.00	+0.02	–0.22	+0.05
Task 12	+0.11	+0.04	+0.06	–0.09	+0.21	–0.42	–0.06
Task 13	–0.10	–0.05	+0.00	+0.08	+0.13	–0.99	+0.01
**Visuospatial attention**							
Task 1	–0.10	–0.59	–0.02	–0.25	+0.14	–0.36	+0.08
Task 2	–0.28	–0.41	–0.03	+0.00	+0.15	–0.37	+0.05
Task 3	–0.33	–0.16	+0.03	–0.18	+0.45	–0.54	+0.01
**Working memory**							
Task 1	–0.12	–0.37	–0.22	–0.06	+0.18	–0.46	+0.04
